# Self-Efficacy Beliefs of Employees with Mental Disorders or Musculoskeletal Diseases after Sickness-Related Absence: Validation of the German Version of the Return-to-Work Self-Efficacy Scale

**DOI:** 10.3390/ijerph191610093

**Published:** 2022-08-15

**Authors:** Marieke Hansmann, Johannes Beller, Friederike Maurer, Christoph Kröger

**Affiliations:** 1 Department of Psychology, University of Hildesheim, Universitätsplatz 1, 31141 Hildesheim, Germany; 2 Medical Sociology, Hannover Medical School, 30625 Hannover, Germany; 3 Department of Psychology, Technical University Brunswick, 38106 Brunswick, Germany

**Keywords:** self-efficacy, return-to-work, vocational rehabilitation, psychotherapy, validation study, psychometrics

## Abstract

Return-to-work self-efficacy (RTW-SE) is an important predictor of the duration until employees return to work after a sickness-related absence. The aim of the present validation study was to investigate the psychometric properties of the German RTW-SE scale. Data were obtained from three independent samples of employees who were in outpatient care due to mental disorders or musculoskeletal diseases (*n*_1_ = 301, *n*_2_ = 103, *n*_3_ = 104). Confirmatory factor analyses showed an inadequate fit for a one-factor solution and an acceptable fit for a two-factor model that distinguished by item-wording direction. To test whether the two factors represent substantively different dimensions of the construct or rather a statistical item-wording effect, two subscales were formed based on item-wording direction. As the subscales were not differentially associated with external measures, the one-factor solution may be considered appropriate. The scale showed good to excellent internal consistency values over time and across samples, had low retest reliability indices, and indicated construct validity based on moderate to high associations with cognitive and disease-related variables. The results further demonstrated the scale’s sensitivity to change. The RTW-SE baseline score predicted physical performance and pain-related psychological impairment after orthopedic rehabilitation. In multiple regression analysis, RTW-SE remained a significant predictor of pain-related psychological impairment but not physical performance, partially demonstrating the predictive validity of the scale. The German version of the RTW-SE scale demonstrated satisfactory results regarding its validity and reliability.

## 1. Introduction

Common mental disorders and musculoskeletal diseases are among the main causes of sickness-related absence in many Western countries [1,2]. Long-term sickness absence is related to low mental well-being [3], a negative development of salary and career possibilities [4], and increasing economic costs due to productivity loss and disability pensions [1]; thus, it constitutes a high burden on the individual and on society. Recent psychotherapeutic approaches and orthopedic rehabilitation aim to reduce symptoms and promote return-to-work (RTW) [5].

The construct of self-efficacy (SE) is an important determinant of the treatment success and the work ability of employees with mental disorders or musculoskeletal diseases [6]. SE refers to the belief in one’s own ability to perform a behavior or task and successfully overcome any difficulties that may arise. People with high SE set higher goals, view tasks as challenges rather than threats, and are more committed to their goals. A distinction is made between a trait-like, generally optimistic, subjective assessment of one’s own coping skills across life domains and independent of situations, and state-like beliefs geared to meeting domain-specific demands such as successfully completing work tasks [7].

A frequently investigated form of domain-specific SE is the return-to-work self-efficacy (RTW-SE). It describes the patient’s belief in being able to cope with the demands of the workplace despite their existing illness [8]. Several studies from the Netherlands have shown that higher RTW-SE beliefs are associated with a shorter duration until a full RTW by employees on sick leave due to mental disorders [9,10], employees on sick leave due to physical problems [11], and long-term sick-listed employees with an all-cause sickness absence [12]. In addition, analyses showed that the RTW-SE of employees with mental disorders or musculoskeletal diseases had a more strongly positive association with the success and duration of RTW than with general SE expectations [13].

These findings underline the relevance of RTW-SE in the context of the development and evaluation of interventions aiming at the promotion of work ability or RTW. The Return-to-Work Self-Efficacy scale is a time-efficient self-evaluation instrument for the assessment of the specific expectations related to RTW [8]. The scale was validated in a population of employees with both mental and physical health problems, and showed excellent values for internal consistency (Cronbach’s α = 0.90 to 0.96) and adequate values for test–retest reliability (*r* = 0.73). It also predicted RTW within three months, and showed increasing RTW-SE levels over the course of psychotherapy, indicating sensitivity to change [8]. So far, there has been no validation of the German version of the RTW-SE scale. Therefore, the aim of the present study was to test the validity and reliability in three different samples of employees with mental disorders or musculoskeletal diseases in Germany. Based on the psychometric properties of the Dutch version, we expected a one-dimensional factorial structure, good internal consistency values, and adequate indices for retest reliability. Moreover, we aimed to add further information on the instrument’s ability to detect clinically important changes by examining various parameters in relation to an external reference standard that has proven to be sensitive to change. As the findings of Lagerveld et al. [8] indicate that the RTW-SE can be used for employees with physical health problems, we further aimed to investigate the predictive value of RTW-SE for physical performance and pain-related psychological impairment after outpatient rehabilitation for employees with musculoskeletal diseases. 

There is a considerable body of empirical research investigating the relationship between SE and mental health outcomes. Study results indicate that higher general and occupational SE are associated with lower levels of depressive symptoms [7,10,14,15], higher life satisfaction [16], and higher job satisfaction in different occupational groups [17,18]. Furthermore, perceived SE can covary between distinct domains when the different realms of activity are determined by similar sub-skills or when extraordinary personal performance (mastery experiences) lead to a restructuring of efficacy beliefs across diverse realms [19]. Based on the above-mentioned findings, we expected negative associations between RTW-SE and levels of depressive symptoms and general symptom burden, and positive associations between RTW-SE and general life satisfaction, satisfaction with work and career, and general SE.

With regard to musculoskeletal diseases, studies have shown that pain-related SE predicted higher lift scores, better physical functioning, and lower disabilities one month after the treatment of patients with chronic back pain [20], and less pain and higher levels of physical activity among patients with osteoarthritis [21]. Due to these results, which point toward the relevance of SE for physical and mental functioning after rehabilitation, we expected that a higher pre-treatment RTW-SE would predict a higher post-treatment physical performance and lower post-treatment pain-related psychological impairment. To determine the relative predictive value of RTW-SE compared to other possible predictors, exploratory analyses were performed.

## 2. Methods

### 2.1. Samples and Procedure

Longitudinal and cross-sectional data were collected in three different convenience samples: (a) a sample of outpatients with different mental disorders who received a clinical psychological assessment and cognitive–behavioral treatment (CBT) in a university outpatient clinic; (b) a sample of outpatients with different mental disorders who participated in an outpatient medical–vocational rehabilitation program due to their psychological impairments; and (c) a sample of outpatients who participated in an orthopedic rehabilitation program due to musculoskeletal diseases. Although the RTW-SE scale was developed to address disability-specific RTW cognitions of employees with mental health problems, the questionnaire showed good psychometric properties in mixed samples of employees who reported either mental or physical health problems [8]. As RTW and the related research is not limited to mental disorders, but also affected by physical diseases [11,12], we aimed to investigate the psychometric properties of the German translation of the RTW-SE scale in samples with both primary mental health problems and primary physical health problems to ensure that the samples of this validation study reflected and captured the range of the target population. Moreover, the samples reflected different treatment programs with regard to patient’s age and length of occupation. 

Participants were recruited by their psychologists in charge from the three public clinics. Questionnaires were administered at the beginning of the treatment (baseline) as part of a standardized data collection for treatment monitoring. For the purpose of investigating test–retest reliability indices, a subset of Sample 1 additionally filled out the RTW-SE scale after a two-week period. Further, questionnaires were administered after the treatment in Samples 1 and 3. Participants were included in the statistical analysis if they were employed or employable (trainees), fluent in German, and provided informed consent. Co-occurring mental disorders or physical diseases were not ruled out. 

#### 2.1.1. Sample 1

The first sample initially consisted of *n* = 360 patients who had completed the RTW-SE scale before intervention. Subsequently, the following patients were excluded: those who refused to participate in the study (*n* = 31), those who had completed the RTW-SE twice at baseline due to a second treatment (*n* = 6), and those who were not in employment (retired, work-seeking, disabled, or housewives/househusbands; *n* = 22). Thus, the data of 301 patients (65.1% male) aged 18 to 62 years (*M* = 41.31, *SD* = 11.37) were included in the analysis. Of the total sample, 30 patients filled out the RTW-SE scale again after a retest interval of two weeks.

Based on cut-off values of the Beck Depression Inventory (BDI-II, German version [22]), more than half of the sample (58.5%) reported a moderate depressive syndrome (BDI-II ≥ 20) (*M* = 23.85, *SD* = 6.69). The most frequent primary diagnoses were affective disorders (30.2%), followed by reactions to severe stress, and adjustment disorders (5.6%). The criteria for at least one co-occurring disorder were met by 17.6% of the sample. Other sample characteristics are listed in Table 1.

This sample was used to investigate the factorial structure, calculate internal consistency and test–retest reliability values, and examine the convergent validity and sensitivity-to-change indices for CBT in a naturalistic setting.

#### 2.1.2. Sample 2

The second sample consisted of 103 patients (57.3% male) aged 17 to 53 years (*M* = 24.24, *SD* = 6.97). Of the total sample, 42 participants (40.8%) were in vocational training, 39 (37.8%) participated in vocational preparation training measures (VPT), and 22 participants (21.4%) were in a rehabilitation program for mentally ill adults (RMA). 

The vocational training and VPT are aimed at adolescents or young adults aged 16 to 25 who do not feel able to cope with the requirements of in-company training as the result of a mental disorder. The RMA includes both medical and vocational rehabilitation measures for adults. The interventions are intended to promote initial entry or re-entry into working life and the general labor market. Participants in the RMA were employed at the rehabilitation center for 4 to 6 h per day, which corresponded to an average of 25 working hours per week. Regular working hours for participants in the VPT were, on average, 36.64 h (*Md* = 38; *SD* = 4.66). The trainees worked 40 h per week. 

Based on the cut-off values of the depression module of the German Patient Health Questionnaire (PHQ-9) [23], almost half of the sample (42.72%) reported moderate depressive symptoms (score ≥ 10) (*M* = 8.97, *SD* = 4.76). Autism spectrum disorders (25.2%) were the most common diagnoses, followed by affective (20.4%) and anxiety disorders (11.7%). The criteria for at least one co-occurring disorder were met by 20 (19.4%) participants. 

Using data from this sample, the convergent validity indices of the RTW-SE scale were examined.

#### 2.1.3. Sample 3

The third sample consisted of 177 patients who participated in an outpatient orthopedic rehabilitation program with the aim of restoring physical performance after orthopedic–traumatological diseases (e.g., joint and spinal diseases, injuries and dysfunction of locomotor organs, condition after surgery, or joint replacement). Patients who experienced a subsequent cure (*n* = 58) and patients with a permanent incapacity to work (*n* = 15) were excluded, so that the data of 104 patients (male = 62.5%) aged 18 to 62 years (*M* = 47.12, *SD* = 9.90 years) were included in the analysis. 

Based on the cut-off values of the depression and anxiety modules of the German PHQ [23], 23.08% reported moderate depressive symptoms (*M* = 6.03, *SD* = 4.83) and 15.38% stated moderate anxiety symptoms (score ≥ 10) (*M* = 4.89, *SD* = 4.57). All participants met orthopedic diagnoses. The most common diagnoses were diseases of the spine and back (61.54%), followed by soft tissue diseases (25.96%), arthropathies (24.04%), and other diseases of the musculoskeletal system and connective tissue (13.46%). Another 12.5% of the sample also met the criteria for mental disorders (46.15% nicotine abuse, 23.08% reactions to severe stress and adjustment disorders, and 23.08% other neurotic disorders). The average duration of treatment was 16 days (*M* = 16.14, *SD* = 2.13).

Using data from this sample, we investigated the predictive value of the baseline RTW-SE for physical performance and pain-related psychological impairment post-treatment.

As expected, between-group comparisons of sample characteristics indicated significant differences concerning age (see Table 1). Dunn–Bonferroni post hoc tests showed that the participants in Sample 2 were significantly younger than the participants in Samples 1 and 3 (*z* = 11.29, *p* < 0.001) and that the participants in Sample 1 were significantly younger than participants in Sample 3 (*z* = −3.88, *p* < 0.001). 

### 2.2. Instruments

#### 2.2.1. Return-to-Work Self-Efficacy (RTW-SE) 

The original version of the Return-to-Work Self-Efficacy scale (RTW-SE scale) [8] consists of 11 items representing expectations with regard to the ability of meeting work demands. Participants are instructed to imagine that they will work their full contract hours tomorrow in their current emotional–cognitive state. The statements are answered on a six-point scale from 1 (“totally disagree”) to 6 (“totally agree”). An example item is: “If I resumed my work fully tomorrow I expect that: I will be able to cope with work pressure.” The internal consistency value of the original version of the scale was excellent (α > 0.90) in various Dutch samples [8].

The items of the German version were translated and back-translated by bilingual experts according to the guidelines for the translation of foreign-language measurement instruments [24], and in cooperation with the Dutch first author, whereby consensus was reached through joint discussions for minor deviations that occurred. 

To perform statistical analysis, first the inverse items (items 2, 6, and 9) were reversed. Then, the RTW-SE scale score was computed by calculating a mean score over all items.

#### 2.2.2. Depressive Symptoms and Anxiety

In Sample 1, the German version of the Beck Depression Inventory (BDI-II) [22] was used to assess depressive symptoms experienced over the past two weeks. The self-report questionnaire consists of 21 items that represent different key symptoms of depression, and are answered on a four-point scale from 0 to 3 with different response categories. Summed scores of 14 or higher indicate clinically significant levels of depressive symptoms. According to Hautzinger et al. [22], the internal consistency values were α = 0.89 to α = 0.93 for various German samples. 

In Samples 2 and 3, symptoms of major depression and symptom severity were measured with the depression module of the German Patient Health Questionnaire (PHQ-9) [23]. The depressive symptoms are assessed regarding their frequency of occurrence during the past two weeks on a four-point scale from 0 (“not at all”) to 3 (“nearly every day”), providing a severity score from 0 to 27. A PHQ-9 sum score of 5 or more indicates mild depressive symptoms, while 10 or more indicates moderate depressive symptoms, 15 or more signifies moderately severe depression, and 20 or more suggests severe depressive symptoms [25]. Further, in Sample 3, symptoms of anxiety were measured with the 7-item anxiety module of the PHQ (GAD-7). The items are evaluated on the same four-point scale as the PHQ-9, resulting in a severity score from 0 to 21 points, whereby a score of 5 or more indicates mild, 10 or more moderate, and 15 or more severe anxiety symptoms [26]. 

Good to excellent internal consistency values could be shown for the depression scale (α = 0.88) and for the anxiety scale (α = 0.92) [27]. 

#### 2.2.3. Overall Psychological Distress

To assess psychological distress in Sample 1, we used the German version of the Symptom Checklist-90-Revised (SCL-90-R) [28]. On 90 items, participants were asked to rate the degree to which they have experienced each of the symptoms during the past 7 days, on a five-point scale from 0 (“not at all”) to 4 (“very much”). The global severity index (GSI), which reflects the overall psychological distress, is obtained by summing the scores of each item and averaging it over the 90 items, whereby a higher GSI indicates a higher symptom burden. In a German-population-representative sample and a German clinical sample, the internal consistency values for the GSI were α = 0.97 in each case [29,30]. 

#### 2.2.4. General Self-Efficacy

The German version of the 10-item General Self-Efficacy scale (GSE scale) [31] was used in Samples 2 and 3 to assess the belief in one’s own competence to master difficult situations and demands. The items represent general SE beliefs (as opposed to domain-specific SE beliefs measured by the RTW-SE scale) and are answered on a four-point scale from 1 (“not at all true”) to 4 (“exactly true”). The internal consistency values in various samples were between α = 0.80 and α = 0.90 [32]. 

#### 2.2.5. Life Satisfaction

With a total of 70 items, the Life Satisfaction Questionnaire [33] measures individual satisfaction in 10 areas of life (e.g., health, work and career, financial situation). The items refer to the past 4 weeks and represent the subjective evaluations of past and present living conditions and future prospects. The items are assessed on a seven-point scale from 1 (“very unsatisfied”) to 7 (“very satisfied”). An index of global life satisfaction is obtained by summing up the values of all subscales except “work and career”, “marriage and partnership” and “relationship with own children”. For the subscale “work and career”, a domain-specific life satisfaction score was calculated. 

The internal consistency values for the subscales ranged from α = 0.82 to α = 0.94, with the “work and career” subscale showing an internal consistency value of α = 0.93 [33].

#### 2.2.6. Physical Performance and Pain-Related Psychological Impairment

Physical performance was assessed by Performance Assessment Capacity Testing (PACT [34]; German version [35]). The self-assessment instrument was developed specifically for people with musculoskeletal diseases. On the basis of fifty items representing daily movements (e.g., “putting dishes in or taking them out of the dishwasher”), the degree to which one is able to perform the corresponding movements is assessed. The items are answered on a five-point scale from 0 (“impossible”) to 4 (“possible”). A high sum score corresponds to a high level of performance. The physical performance at the end of rehabilitation can be regarded as an indicator of the physical aspects of rehabilitation success. 

The Pain-Processing Questionnaire (PPQ) [36] (for the detection of pain-coping strategies and pain-related distress) was used to assess pain-related psychological impairments. This dimension of pain processing is evaluated by the three subscales “pain-related helplessness and depression”, “pain-related fear”, and “pain-related anger”. The fourteen items are answered on a six-point scale from 1 (“do not agree at all”) to 6 (“completely agree”). A high sum score over the three scales corresponds to a high level of impairment. The pain-related psychological impairment can be regarded as an indicator for the psychological aspect of rehabilitation success, because pain represents a significant aspect of illness in the primary diagnoses of this sample. 

In the present study, the internal consistency values were acceptable to excellent for all instruments (see Table S1, electronic Supplementary Materials).

### 2.3. Statistical Analysis

Statistical analyses were conducted with the IBM statistical software SPSS (version 27). Confirmatory factor analyses (CFAs) were performed using the lavaan package for R statistics. 

#### 2.3.1. Missing Data

The proportion of missing values for the items of the RTW-SE scale was between 0% (Samples 2 and 3) and 28.39% (Sample 1) at baseline. To account for missing data, multiple imputation was applied to create and analyze 40 multiply imputed datasets. As recommended by Heymans and Eekhout [37], the estimated values were corrected by predictive mean matching to avoid implausible item values. 

#### 2.3.2. Factorial Validity

To test the proposed one-dimensional structure of the RTW-SE scale, CFAs were conducted for a one-factor model and a two-factor model, with one factor representing positively worded (PW) items and one factor representing negatively worded (NW) items. A maximum likelihood estimation with robust standard errors and a Satorra–Bentler scaled test statistic was used. This robust statistic is considered to protect against distortions of Type I error rates due to nonnormality and is recommended for use with small sample sizes [38,39]. Several fit indices were calculated to test the appropriateness of the model: the comparative fit index (CFI), the Tucker–Lewis index (TLI), and the root mean square error of approximation (RMSEA). According to Hu and Bentler [40], CFI and TLI values > 0.95 represent a good model fit. RMSEA values < 0.05 indicate a good fit and values < 0.10 represent an acceptable fit [41]. The discriminant validity of the factors in the two-factor model was assessed by (a) comparing Pearson’s correlation coefficients of the PW and the NW subscale with external measures and (b) comparing the average variance extracted (AVE) and the correlation coefficient square of the two factors. 

#### 2.3.3. Convergent and Discriminant Validity

The convergent and discriminant validity indices were assessed by calculating Pearson’s correlation coefficients between the baseline scores of the RTW-SE scale and the following self-report instruments: the GSE scale, the BDI-II, the SCL-90-R, the global score, and the “work and career” subscale score of the LSQ.

#### 2.3.4. Predictive Validity

The predictive value of the RTW-SE scale was studied by analyzing the longitudinal relations between the baseline RTW-SE and two outcome measures of orthopedic rehabilitation: (a) physical performance and (b) pain-related psychological impairment after treatment. First, simple linear regression analyses were performed for each criterion. In a second step, multiple linear regression analyses were performed to investigate the relative predictive value of RTW-SE in comparison to other predictors.

Due to the heterogeneous results of predictor research in orthopedic rehabilitation, Farin et al. [42] proposed the inclusion of sociodemographic, psychosocial, and disease- and treatment-specific predictors, as well as the baseline values of the examined criteria in the prediction model. Therefore, the initial multiple regression models included the following predictors: age, gender, occupational status, RTW-SE, depressiveness, anxiety, and baseline values of physical performance and pain-related psychological impairment. 

#### 2.3.5. Reliability

The internal consistency values of the RTW-SE scale are reported as Cronbach’s alpha and McDonald’s omega. The retest reliability estimate of the RTW-SE scale was studied within Sample 1 (*n* = 30) by calculating Spearman’s correlation coefficient between the baseline measurement and after a two-week follow-up period.

#### 2.3.6. Sensitivity to Change

In accordance with the recommendations for assessing the sensitivity-to-change indices in a single-group design [43], various parameters were calculated. Pre–post mean comparisons of the RTW-SE scale were conducted using dependent *t*-tests. To quantify the magnitude of change, pooled effect sizes were calculated (*d* = M_1_ − M_2_/√(SD_1_^2^ + SD_2_^2^/2)). Moreover, the pre–post mean differences of the RTW-SE scale were correlated with the pre–post mean differences of the BDI-II (external criterion), which is known to be sensitive to change [44]. Further, the internal consistency values of the RTW-SE scale were specified for pre- and post-measurement.

Using the formula recommended by Jacobson and Truax [45], the percentages of patients whose depressive symptoms and RTW-SE reliably improved, worsened, or remained unchanged according to the respective instrument were determined. For this purpose, normative data and reliability coefficients were derived from normative samples of the RTW-SE (*M* = 3.27, *SD* = 1.31, α = 0.95) [8] and the BDI-II (*M* = 12.9, *SD* = 9.6, α = 0.89; sample of (partially) remitted patients) [22]. To determine the percentage of patients whose test scores shifted from the dysfunctional to the functional population, cut-off scores were applied. According to Hautzinger et al. [22], a BDI-II score of ≥ 19 represents a reliable cut-off score. For RTW-SE, the cut-off point was calculated as the sum of the mean pre-treatment score of outpatients and their standard error: M_Dys_ + SE = 3.27 + (1.31[1 − 0.95]^1/2^) = 3.56. Individuals who reliably improved and fell below (BDI-II) or above (RTW-SE) the cut-off value were considered to have shown clinically significant improvement.

## 3. Results

### 3.1. Descriptive Statistics

Table S2 displays the means and standard deviations for each item of the RTW-SE scale for the three samples. The skewness indices for the original (before imputation) RTW-SE baseline data ranged from −0.44 to 0.01. The kurtosis indices ranged from −0.65 to 0.05 (see Table S3). The distribution of the baseline RTW-SE was different across samples (Welch’s *F*(2, 238.638) = 31.67, *p* < 0.001, η^2^ = 0.11). The means ranged from 3.68 to 4.49, while the standard deviations ranged from 0.82 to 1.17 (see Table 2). Patients who received outpatient psychotherapy had lower baseline RTW-SE scores than patients who participated in an outpatient medical–vocational rehabilitation due to mental disorders or outpatient orthopedic rehabilitation, whereas the mean RTW-SE scores of the latter samples did not differ significantly.

### 3.2. Factorial Validity

To test whether our data were suited for a CFA, the Kaiser–Meyer–Olkin measure was calculated (KMO = 0.94). The Bartlett’s test of sphericity indicated that the correlations between items were sufficient for conducting a CFA (χ^2^(55) = 95,702.1, *p* < 0.001). 

The one-dimensional model showed an inadequate fit, with RMSEA = 0.102 [95% CI: 0.100–0.105], TLI = 0.873, and CFI = 0.899. Based on assumptions of method effects in the self-report measures, with a mix of both PW and NW items causing an inadequate model fit [46], we tested whether a two-dimensional model, consisting of one factor representing PW items and one representing NW items, fit the data better. Indeed, all indices showed an acceptable to good fit for the two-factor model distinguishing by item-wording directions, with RMSEA = 0.069 [95% CI: 0.067–0.071], TLI = 0.942, and CFI = 0.955. The first- and second-order-factor loadings are displayed in Table S4. 

Based on these results, we formed two subscales distinguishing between PW items (items 1, 3, 4, 5, 7, 8, 10, and 11) and NW items (items 2, 6, and 9) and examined their relation to the external measures separately to test whether the two factors of the scale were substantially meaningful or rather a methodological artifact [46,47]. The *t*-statistic for the difference in correlations was computed as proposed by Chen and Popovich [48] and checked against the appropriate critical value for *t* with *N*-3 degrees of freedom. The critical values are 1.98 (*p* < 0.05) and 2.63 (*p* < 0.01), two-tailed. Although the correlations between the PW subscale and the GSE scale were higher (Sample 2: *r* = 0.56; *p* < 0.001; Sample 3: *r* = 0.50, *p* < 0.001) than the correlations between the NW subscale and the GSE scale (Sample 2: *r* = 0.30, *p* = 0.002; Sample 3: *r* = 0.38, *p* < 0.001; see Table 3), the coefficients did not differ regarding the direction of the association. A comparison of the correlation coefficients of the two subscales with other external measures did not show any further significant differences between the PW and the NW subscales. Moreover, a discriminant validity analysis showed that the AVE of the second factor was not substantively higher (AVE_factor1_ = 0.657, AVE_factor2_ = 0.526) than the squared correlation coefficient of the two factors (ρ^2^ = 0.53), indicating that the two factors do not represent distinct constructs. Thus, the better fit of the two-factor model may be regarded as an artifact resulting from method effects related to the item-wording direction rather than a substantially meaningful two-factorial structure of the construct. Therefore, the one-factor solution was chosen for further analyses. 

### 3.3. Convergent and Discriminant Validity

Correlation coefficients between the RTW-SE scale and several measures are shown in Table 3. Higher levels of RTW-SE were moderately to strongly associated with a higher GSE (Sample 2: *r* = 0.54, *p* < 0.001; Sample 3: *r* = 0.51, *p* < 0.001) and higher satisfaction with work and career (*r* = 0.46, *p* < 0.001). Moreover, higher RTW-SE was moderately correlated with lower depression levels (*r* = −0.36, *p* < 0.001), higher general life satisfaction (*r* = 0.35, *p* < 0.001), and lower general symptom burden (*r* = −0.29, *p* < 0.001).

### 3.4. Predictive Validity 

As shown in Table S5, age and occupational status were not associated with the criterion variables (*p* > 0.05); hence, we removed these sociodemographic variables from regression analyses. Gender was only included in the regression model for physical performance. The predictor variables were entered simultaneously into the models (enter method).

There was no multicollinearity in our data, as revealed by VIF scores below 5 and tolerance scores above 0.2. The values of the residuals were normally distributed, and the Durbin–Watson test showed values between 1.90 and 2.10, indicating that the values of the residuals were independent. According to recommendations concerning significance tests and estimates for *R*^2^ in multiply imputed datasets [49], the standardized regression coefficients (β) and *R*^2^ were averaged across imputed datasets.

After the 3-week rehabilitation program, patients reported higher physical performance and lower pain-related psychological impairment compared to the pre-intervention level (see Table 4). 

Linear regression analyses showed that a higher baseline RTW-SE was a predictor of higher physical performance (*β* = 0.33, *T* = 3.56, 95% CI: 6.18–21.38, *p* < 0.001) and lower pain-related psychological impairment after treatment (*β* = −0.45, *T* = −5.03, 95% CI: −10.75 to −4.72, *p* < 0.001). RTW-SE explains 11.13% of the total variance in physical performance (*R*^2^ = 0.11, *p* < 0.001) and 20.07% of the total variance in pain-related psychological impairment (*R*^2^ = 0.20, *p* < 0.001).

The results of the multiple regression analyses are presented in Table 5. The model for physical performance was statistically significant (*F*(5, 98) = 27.72; *p* < 0.001; *R*^2^ = 0.59). Of all predictors, only the pre-intervention level of physical performance was found to be a significant predictor of post-treatment physical performance. The model for pain-related psychological impairment was statistically significant (*F*(4, 99) = 15.15; *p* < 0.001; *R*^2^ = 0.38). Pain-related psychological impairment after treatment was predicted by the baseline level of pain-related psychological impairment and RTW-SE (*p* < 0.05).

### 3.5. Reliability 

Cronbach’s alpha and McDonald’s omega are presented in Table S6. The RTW-SE scale showed good to excellent internal consistency values over time and across samples. 

Spearman’s correlation coefficient for the association between the baseline measurement and the two-week follow-up was *r_s_* = 0.43 (*p* = 0.017).

### 3.6. Sensitivity to Change

Pre–post mean comparisons of the RTW-SE scale and the BDI-II showed that RTW-SE significantly increased and depressive symptoms significantly decreased after outpatient psychotherapy (see Table 6). Although the extent to which depressive symptoms were reduced was larger than the growth in RTW-SE, both changes showed large effect sizes [50]. The moderate association of the mean differences of the RTW-SE and the BDI (*r* = −0.31; *p* < 0.001) indicated that the change depicted in the BDI-II, which is considered to be change-sensitive, can be found in the RTW-SE scale.

The high internal consistency value of the RTW-SE (α_pre_ = 0.93) was maintained after treatment (α_post_ = 0.82). However, the rates of change differed depending on the instrument (χ^2^ (2) = 19.90; *p* < 0.001). According to the BDI-II, more patients were reliably improved than according to RTW-SE (see Table 6). After treatment, 290 patients (96.35%) showed a level of depressive symptoms that corresponded to that of a (partially) remitted group of outpatients. A similar number of patients (*n* = 283; 94.02%) exceeded the cut-off score and showed RTW-SE levels higher than those of outpatients. Further, more patients can be considered to show clinically significant improvement with regard to depressive symptoms than with regard to RTW-SE.

## 4. Discussion

The aim of this study was to investigate validity and reliability indices of the German translation of the Return-to-Work Self-Efficacy (RTW-SE) scale within three independent samples of employees who were in outpatient care due to mental disorders or musculoskeletal diseases.

The questionnaire showed moderate to high correlation coefficients with several indicators of construct validity, and predicted the rehabilitation outcome in an outpatient orthopedic setting. Moreover, the scale showed good to excellent internal consistency values across samples, indicating that it can be applied in different settings. Further, it was sensitive to changes over the course of outpatient psychotherapeutic treatment. 

### 4.1. Factorial Validity

A confirmatory factor analysis was conducted to investigate whether the factor structure of the German RTW-SE scale corresponds to the proposed one-dimensional structure. Contrary to the findings of Lagerveld et al. [8], the assessed indices showed an inadequate fit for the one-dimensional model. However, for methodological reasons, the single-factor solution is preferred: First, our finding is in line with previous research indicating that one-dimensional factor models do not provide an adequate fit for self-report measures that contain a mix of PW and NW items, because reverse-scoring can produce a method factor that does not represent a substantially distinct dimension of the construct [46,47,51]. Second, we tested (a) whether the PW subscale and NW subscale of the RTW-SE scale were differentially related to the external criteria (indicators of construct validity) and (b) whether the AVE was higher than the correlation coefficient square of the two factors to test whether the two factors are substantially meaningful. We found higher positive correlations between the PW subscale and general self-efficacy (GSE) than between the NW subscale and GSE. However, there was no difference in the direction of the correlation. With regard to the remaining criteria, there was no significant differentiation between the correlation coefficients based on the positive and the negative factors. Moreover, AVE was effectively equal to the correlation coefficient square of the two factors; hence, discriminant validity results indicate that the two-factor model does not represent two distinct dimensions of the construct, but rather a method effect without substantial meaning. Third, the NW subscale comprises only three items. To measure a negative dimension of the RTW-SE construct adequately, a more detailed operationalization would be needed. Therefore, the single-factor solution may be considered appropriate.

### 4.2. Predictive Value for Physical Performance and Pain-Related Psychological Impairment

A higher baseline RTW-SE predicted higher physical performance and lower pain-related psychological impairment. However, RTW-SE did not remain a significant predictor of physical performance after treatment when other possible predictors (depressive and anxiety symptoms) were considered. This finding might be explained by the usually high association between self-efficacy expectations and depressive complaints [7,15].

Earlier studies suggest a prognostic value of RTW-SE and how it changed during CBT for the duration until full RTW, which remained significant even when controlling for symptom improvement [9,10]. Our study shows that RTW-SE might additionally be a relevant predictor for pain-processing in orthopedically impaired employees. However, the prognostic value of RTW-SE for the subjective judgement of physical performance after treatment does not exceed the prognostic value of psychological symptoms. A variety of other health-related or cognitive–behavioral risk factors might play a role in the prediction of physical performance in patients with musculoskeletal pain (e.g., external locus of control, higher levels of anxiety [52,53]). 

### 4.3. Reliability

The internal consistency values were excellent across samples and remained excellent at a two-week follow up. However, the retest reliability estimate was relatively low. This result is in line with the findings of Lagerveld et al. [8], who reported a lower retest reliability value (*r* = 0.47) in a subsample with clinical mental health disorders compared to a mixed sample with mental and physical health problems. Similarly, Kühner et al. [44] reported a lower retest reliability value for depressive symptoms assessed by the BDI-II in a clinical sample (*r* = 0.47) compared to a non-clinical sample (*r* = 0.78). The low stability of RTW-SE in employees with mental health disorders reflects the nature of a state characteristic and might indicate a differential, non-monotonic reactivity to events; for example, a higher variability in the degree or duration of changes over time in employees with mental health disorders compared to employees without a mental health diagnosis. In light of the fact that CBT aims at enhancing the SE of patients, a low stability is considered appropriate because it ensures the desired modification of RTW-SE. However, a thorough empirical investigation is needed to address the question of potential population-dependent differences in the stability of RTW-SE.

### 4.4. Sensitivity to Change

The high reliability value of the RTW-SE scale was stable over time, indicating, for example, that the means and standard deviations are allowed to be calculated. The large effect size of the determined RTW-SE increase corresponds to the degree of RTW-SE increase after six months of psychotherapy in the original Dutch sample (with self-calculated ES = 0.93 from Sample 2) [8]. The improvement that occurred after treatment is reflected in the BDI-II, which is known to be sensitive to change [22], and for which the change scores showed a moderate correlation with the RTW-SE change scores. However, the BDI-II showed a higher proportion of both reliably improved and clinically stabilized patients than the RTW-SE scale (60% vs. 50%). 

This post-treatment reduction in RTW-SE for 10% of the sample might lead to the conclusion that CBT was not successful in improving RTW-SE for some of the patients. However, SE judgements are considered to be dynamic, and declines occur when difficulties are encountered or when problems are focused [54,55,56]. It might be that the patients were faced with work-related problems that influenced their judgements of RTW-SE at the time of post-measurement. A study showing that a learner’s self-efficacy varied reliably multiple times over observations within a single learning task indicated that pre–post assessments cannot accommodate non-monotonic intra-individual change and are therefore not sufficient for drawing conclusions about the effect of psychotherapy on self-efficacy [57].

RTW-SE is a relevant predictor of the probability and duration until RTW after a sickness-related absence due to mental disorders [6]. Therefore, the dynamic nature of RTW-SE in samples of employees with mental complaints should be investigated in more detail in future studies to understand the specific factors that cause intra-individual changes of RTW-SE. If daily events (e.g., conversations with colleagues) or specific experiences (e.g., consultation of occupational reintegration management) are proven to contribute to significant changes—especially improvements—in RTW-SE, these factors should be addressed in psychotherapeutic treatment planning [58].

Including an external criterion allowed us to extend the findings of Lagerveld et al. [8] that resulted from a single-measure pre–post mean comparison. Therefore, our results contribute to a better understanding of the questionnaire’s ability to detect clinically important changes in RTW-SE, and support the notion that the instrument might not only be suitable for prognostic research questions, but also for the measurement of change.

### 4.5. Limitations and Implications for Future Research

The present study encompasses some limitations that need to be addressed. The data were obtained by self-assessment, and we did not collect any data from sources other than the individual report, e.g., sickness days listed in registration systems of the health insurance companies. Thus, the objectivity of the results is restricted. 

Concerning the factorial structure, it should be taken into account that method factors due to item wording direction can represent a type of response style that is associated with intrapersonal characteristics (e.g., life satisfaction, neuroticism) [46,47]. Further research is needed to gain a better understanding of the factorial structure of the scale and the underlying determinants of a two-factor model (substantively irrelevant artifact vs. type of response style in some participants). Further, an analysis of invariance by age group, gender, or country of origin (cross-cultural invariance) might be interesting.

Although the results add information on the predictive value of RTW-SE, it should be noted that no psychosocial predictors except gender were included in the multiple regression models due to a lack of existing correlations between age, occupational status, and the criterion variables. Therefore, future studies should investigate the context-specific relative predictive value of RTW-SE for different indicators of physical and mental health, by including a broader variety of psychosocial, disease-specific, and treatment-specific predictors, as well as the baseline values of the examined criteria in the prediction model [42].

Concerning the results of the sensitivity-to-change analysis, it should be mentioned that the external criterion measure (BDI-II) did not correspond to aa gold standard that captures the same construct like the RTW-SE scale (i.e. work-related or specific SE beliefs). Therefore, the generalizability of our results is limited such that a relative sensitivity to change in relation to a sensitive symptom measure of depression can be assumed. Further, no assumptions can be made about the degree to which the intervention contributed to the improvement of RTW-SE, as no (waitlist) control group was included in our study. Future studies should administer a baseline measurement interval before the onset of therapy, so as to investigate random or event-related changes in RTW-SE, thus taking into account the presumed fluctuating nature of specific SE expectations. Further, to our knowledge, the questionnaire has never been administered to samples of healthy employees who lack work-related problems. Therefore, it is unclear whether RTW-SE reached a level of the occupational SE beliefs of healthy employees. 

## 5. Conclusions

This study investigated the factorial structure, convergent validity, predictive validity, reliability, and sensitivity to change in three independent German samples of employees with mental disorders or musculoskeletal diseases. The present findings add valuable information to the psychometric properties of the RTW-SE scale.

## Figures and Tables

**Table 1:** Sample characteristics.

	Sample 1 *N* = 301	Sample 2 *N* = 103	Sample 3 *N* = 104	Difference
*M* age (*SD*)	41.31 (11.37)	24.24 (6.97)	47.12 (9.90)	χ^2^ (2) = 176.45; *p* < 0.001 ^a^
Gender (% male)	196 (65.1)	59 (57.3)	65 (62.5)	χ^2^ (2) = 2.034; *p* = 0.362 ^b^
Higher education (% high school graduation)	73 (24.3)	11 (10.6)	–	
*Social status (%)*				
Single	88 (29.2)	97 (94.1)	26 (25.0)	
Married/in a relationship	178 (59.2)	4 (3.9)	67 (64.4)	
Divorced	23 (7.6)	1 (1.0)	11 (10.6)	
Widowed	3 (1.0)	1 (1.0)	0	
NA	9 (3.0)	0	0	
*Vocational education (%)*				
Apprenticeship	162 (53.8)	–	77 (74.0)	
Vocational academy (e.g., master school)	–	–	10 (9.6)	
College/university	20 (6.6)	–	10 (9.6)	
Currently undergoing training or studying	16 (5.3)	–	–	
Other vocational training	–	–	4 (3.9)	
No completed vocational training	5 (1.7)	–	3 (2.9)	
NA	98 (32.6)	–	0	
*Vocational status (%)*				
Qualified specialist	141 (46.8)	–	–	
Employee or civil servant	131 (43.5)	–	–	
Self-employed	3 (1.0)	–	–	
Currently undergoing (re)training	18 (6.0)	–	–	
NA	8 (2.7)	–	–	

Note: NA = not available (missing value); ^a^ = Kruskal–Wallis test, ^b^ = Pearson’s chi-squared test.

**Table 2:** Means and standard deviations of the RTW-SE scale.

	Sample 1	Sample 2	Sample 3
	*M* (*SD*)	*M* (*SD*)	*M* (*SD*)
Baseline	3.68 (1.17)	4.37 (0.82)	4.49 (0.96)
After two weeks	3.93 (0.99)	–	–
Post	4.50 (0.72)	–	4.65 (0.90)

**Table 3:** Correlation coefficients for associations between (1) baseline RTW-SE, (2) PW, and (3) NW subscales and cognitive and disease-related variables.

	RTW-SE		PW		NW		PW–NW		Mean Comparison	
	*r*	*p*	*r*	*p*	*r*	*p*	*r*	*p*	T	*p*
GSE ^b^	0.54	<0.001	0.56	<0.001	0.30	0.002	0.54	<0.001	4.341	<0.01
GSE ^c^	0.51	<0.001	0.50	<0.001	0.38	<0.001	0.60	<0.001	2.211	<0.05
BDI ^a^	−0.36	<0.001	−0.34	<0.001	−0.31	<0.001	0.62	<0.001	−0.481	>0.05
SCL ^a^	−0.29	<0.001	−0.29	<0.001	−0.21	<0.001	0.62	<0.001	−1.375	>0.05
LSQ ^a^	0.35	<0.001	0.32	<0.001	0.32	<0.001	0.62	<0.001	0	>0.05
LSQ work ^a^	0.46	<0.001	0.44	<0.001	0.39	<0.001	0.62	<0.001	1.570	>0.05

Note: ^a^ = Sample 1, ^b^ = Sample 2, ^c^ = Sample 3. PW = RTW-SE subscale of positively worded items, NW = RTW-SE subscale of negatively worded items. PW–NW = correlation between the PW and NW subscales.

**Table 4:** Means and standard deviations of physical performance (PACT) and pain-related psychological impairment (PPQ) before and after outpatient orthopedic rehabilitation and effect sizes.

	Baseline		Post		Mean Comparison		
	*M*	*SD*	*M*	*SD*	T	*p*	ES
PACT	114.87	39.26	128.88	39.73	−5.03	<0.001	0.35
PPQ	39.38	15.81	33.06	16.65	3.98	<0.001	0.39

**Table 5:** Results of multiple regression analysis with the criteria of physical performance and pain-related psychological impairment after treatment.

	*B*	*SE B*	*β*	95% CI for *B*	*t*	*p*
Physical performance after treatment (PACT_post_)						
Gender	5.58	5.80	0.07	−5.78, 16.95	0.96	0.336
RTW-SE_pre_	0.81	3.51	0.02	−6.08, 7.69	0.23	0.819
PHQ-9_pre_	−0.88	0.91	−0.11	−2.66, 0.90	−0.97	0.333
GAD-7_pre_	−0.34	0.98	−0.04	−2.26, 1.58	−0.35	0.727
PACT_pre_	0.69	0.07	0.68	0.55, 0.83	9.68	<0.001
Pain-related psychological impairment after treatment (PPQ_post_)						
RTW-SE_pre_	−3.46	1.74	−0.20	−6.87, −0.06	−1.99	0.046
PHQ-9_pre_	−0.10	0.47	−0.01	−1.03, 0.83	−0.22	0.827
GAD-7_pre_	0.94	0.49	0.26	−0.03, 1.91	1.91	0.056
PPQ_pre_	0.36	0.10	0.34	0.17, 0.55	3.71	<0.001

Note: *B* = unstandardized beta; *SE* = standard error of beta; *β* = standardized beta; 95% CI = 95% confidence interval. RTW-SE = return-to-work self-efficacy; PHQ-9 = depression module of the Patient Health Questionnaire; GAD-7 = anxiety module of the Patient Health Questionnaire.

**Table 6:** Means, standard deviations, effect sizes, and clinical significance before and after outpatient psychotherapy.

	Baseline		Post		Mean Comparison			Clinical Significance (%)			
	*M*	*SD*	*M*	*SD*	*T*	*p*	ES	ci	ri	rd	nc
RTW-SE	3.68	1.17	4.50	0.72	−11.06	<0.001	0.84	150 (49.83)	155 (51.50)	31 (10.30)	115 (38.20)
BDI	23.85	6.69	12.75	4.36	25.62	<0.001	1.97	180 (59.8)	184 (61.13)	-	117 (38.87)

Note: ES = effect size, ci = clinically improved, ri = reliably improved, rd = reliably deteriorated, nc = not reliably changed.

## Data Availability

The datasets analyzed during the current study are available from the corresponding author upon reasonable request.

## Supplementary Materials

The following supporting information can be downloaded at: https://www.mdpi.com/article/10.3390/ijerph191610093/s1, Table S1: Internal consistency values for all instruments of the present study. Table S2: Means and standard deviations of the RTW-SE items. Table S3: Skewness and kurtosis indices for scale scores of original baseline data. Table S4: Factor loadings of the RTW-SE items for the one-factor solution and the two-factor solution (standardized regression coefficients). Table S5: Correlations coefficients between physical performance (PACT)/pain-related psychological impairment (PPQ) and RTW-SE, symptom measures, and sociodemographic variables. Table S6: Internal consistency values of the RTW-SE scale.
