# Supplementary material for: Self-Efficacy Beliefs of Employees with Mental Disorders or Musculoskeletal Diseases after Sickness-Related Absence: Validation of the German Version of the Return-to-Work Self-Efficacy Scale
Source: Int J Environ Res Public Health. 2022 Aug 15;19(16):10093. doi: 10.3390/ijerph191610093 (PMC9408298; doi:10.3390/ijerph191610093)
Supplement: Supplementary file 1 [file ijerph-19-10093-s001.zip › ijerph-1832305-supplementary.pdf]

Self-efficacy beliefs of employees with mental disorders or musculoskeletal diseases after sickness-related absence: Validation of the German version of the Return-to-Work Self-Efficacy Scale

International Journal of Environmental Research and Public Health

Supplementary Material

Marieke Hansmann,[a]* Johannes Beller,[b] Friederike Maurer,[c] & Christoph Kröger[a]

[a]*Department of Psychology, University of Hildesheim, Hildesheim, Germany;* [b]*Medical Sociology, Hannover Medical School, Hannover, Germany;* [c]*Department of Psychology, Technical University Brunswick, Brunswick, Germany*

*Corresponding author: Universitätsplatz 1, 31141 Hildesheim; email: marieke.hansmann@uni-hildesheim.de; Telephone: +495121 883 11070

Table S1. Internal consistency values for all instruments of the present study.

	Sample 1	Sample 2	Sample 3
	Alpha	Alpha	Alpha
BDI-II	.89	–	–
PHQ-9	–	.76	.84
GAD-7	–	–	.88
SCL-90-R	.97	–	–
GSE	–	.88	.90
LSQ global	.93	–	–
LSQ work	.85	–	–
PACT	–	–	.98
PPQ	–	–	.92

Note: BDI-II = Beck-Depression-Inventory II, PHQ-9 = Depression module of the Patient Health Questionnaire, GAD-7 = Anxiety module of the Patient Health Questionnaire, SCL-90-R = Symptom-Checklist-90-Revised, GSE = General Self-Efficacy Scale, LSQ work = "work and career" subscale of the Life Satisfaction Questionnaire, PACT = Performance Assessment Capacity Testing, PPQ = Pain processing questionnaire.

Table S2. Means and standard deviations of the RTW-SE items.

	Sample 1		Sample 2		Sample 3	
	M	*SD*	*M*	*SD*	*M*	*SD*
(1) I will be able to cope with setbacks.	3.50	1.60	4.19	1.19	4.42	1.26
(2) I won't be able to complete my work tasks due to my emotional state.*	3.60	1.60	4.24	1.31	4.57	1.46
(3) I will be able to set my personal boundaries at work.	3.78	1.46	4.45	1.14	4.45	1.28
(4) I will be able to perform my tasks at work.	4.15	1.45	5.04	.98	4.63	1.25
(5) I will be able to deal with emotionally demanding situations.	3.49	1.52	4.10	1.13	4.54	1.26
(6) I will have no energy left to do anything else.*	3.45	1.59	3.95	1.38	4.30	1.47
(7) I will be able to concentrate on my work.	3.66	1.50	4.42	1.29	4.60	1.21
(8) I will be able to cope with work pressure.	3.53	1.58	4.32	1.37	4.41	1.45
(9) I won't be able to handle potential problems at work.*	3.65	1.54	4.05	1.24	4.68	1.31
(10) I can motivate myself to perform my job.	3.71	1.51	4.60	1.15	4.64	1.35
(11) I can deal with the physical demands of my work.	3.99	1.48	4.70	1.15	4.17	1.54

Note: * reversed items.

Table S3. Skewness and kurtosis indices for scale scores of original baseline data.

	**Sample 1**		**Sample 2**		**Sample 3**	
	Skewness	*Kurtosis*	*Skewness*	*Kurtosis*	*Skewness*	*Kurtosis*
RTW-SE	.01	-.65	-.44	.05	-.43	-.41
GSE	–	–	-.12	.34	-.23	-.16
BDI	.26	-.46	–	–	–	–
SCL	.88	.84	–	–	–	–
LSQ	-.02	-.45	–	–	–	–
LSQ work	-.16	-.70	–	–	–	–
PHQ-9	–	–	.51	-.14	.84	-.02
GAD-7	–	–	–	–	1.04	.13
PACT	–	–	–	–	.06	-.05
PPQ	–	–	–	–	.51	-.06

Table S4. Factor loadings of the RTW-SE items for the one-factor solution and the two-factor solution (standardized regression coefficients)

	One-dimensional	Two-dimensional	
	RTW-SE factor	Factor 1 (PW)	Factor 2 (NW)
Item 1	.77***	.77***	
Item 2	.54***		.70***
Item 3	.64***	.64***	
Item 4	.85***	.86***	
Item 5	.84***	.84***	
Item 6	.52***		.71***
Item 7	.88***	.88***	
Item 8	.89***	.89***	
Item 9	.58***		.77***
Item 10	.83***	.83***	
Item 11	.72***	.73***	

Note: RTW-SE = Return-to-work self-efficacy scale; PW = RTW-SE subscale of positively worded items, NW = RTW-SE subscale of negatively worded items. *** p < .001

Table S5. Correlation coefficients between physical performance (PACT)/pain-related psychological impairment (PPQ) and RTW-SE, symptom measures and sociodemographic variables.

	PPQ_{post}		$PACT_{post}$	
	r	*p*	*r*	*p*
PPQ_{pre}	.51	< .001	-.24	.017
$PACT_{pre}$	-.28	.004	.75	< .001
$RTW\text{-}SE_{pre}$	-.45	< .001	.33	.001
$PHQ\text{-}9_{pre}$	.45	< .001	-.34	< .001
$GAD\text{-}7_{pre}$	.50	< .001	-.35	< .001
Age	-.07	.509	-.15	.133
Gender	.06[a]	.500	.29[a]	.003
Occupational status	.16[a]	.752	.22[a]	.441

Note: RTW-SE = Return-to-work self-efficacy; PHQ-9 = Depression module of the Patient-Health Questionnaire; GAD-7 = Anxiety module of the Patient-Health-Questionnaire. [a] Eta for the description of association between a nominal and a metric variable.

Table S6. Internal consistency values of the RTW-SE scale.

	Sample 1		Sample 2		Sample 3	
	Alpha	Omega t	Alpha	Omega t	Alpha	Omega t
Baseline	.93	.95	.88	.91	.90	.93
Two weeks	.92	.96	–	–	–	–
Post	.82	.84	–	–	.89	.92

Note: Omega t = Omega total.
